# Supplementary material for: Baseline Characterization of the Gut Microbiota of Field and Colony Populations of Phlebotomus tobbi and Preliminary Assessment of the Anti-Leishmanial Activity of Cultivable Bacteria
Source: Pathogens. 2026 Jun 23;15(7):658. doi: 10.3390/pathogens15070658 (PMC13416292; doi:10.3390/pathogens15070658)
Supplement: Supplementary file 1 [file pathogens-15-00658-s001.zip › pathogens-4357168-File S1/Taxa2ASV_Decomposer/Family/f__Pseudomonadaceae/heatmap_with_clustering.pdf]

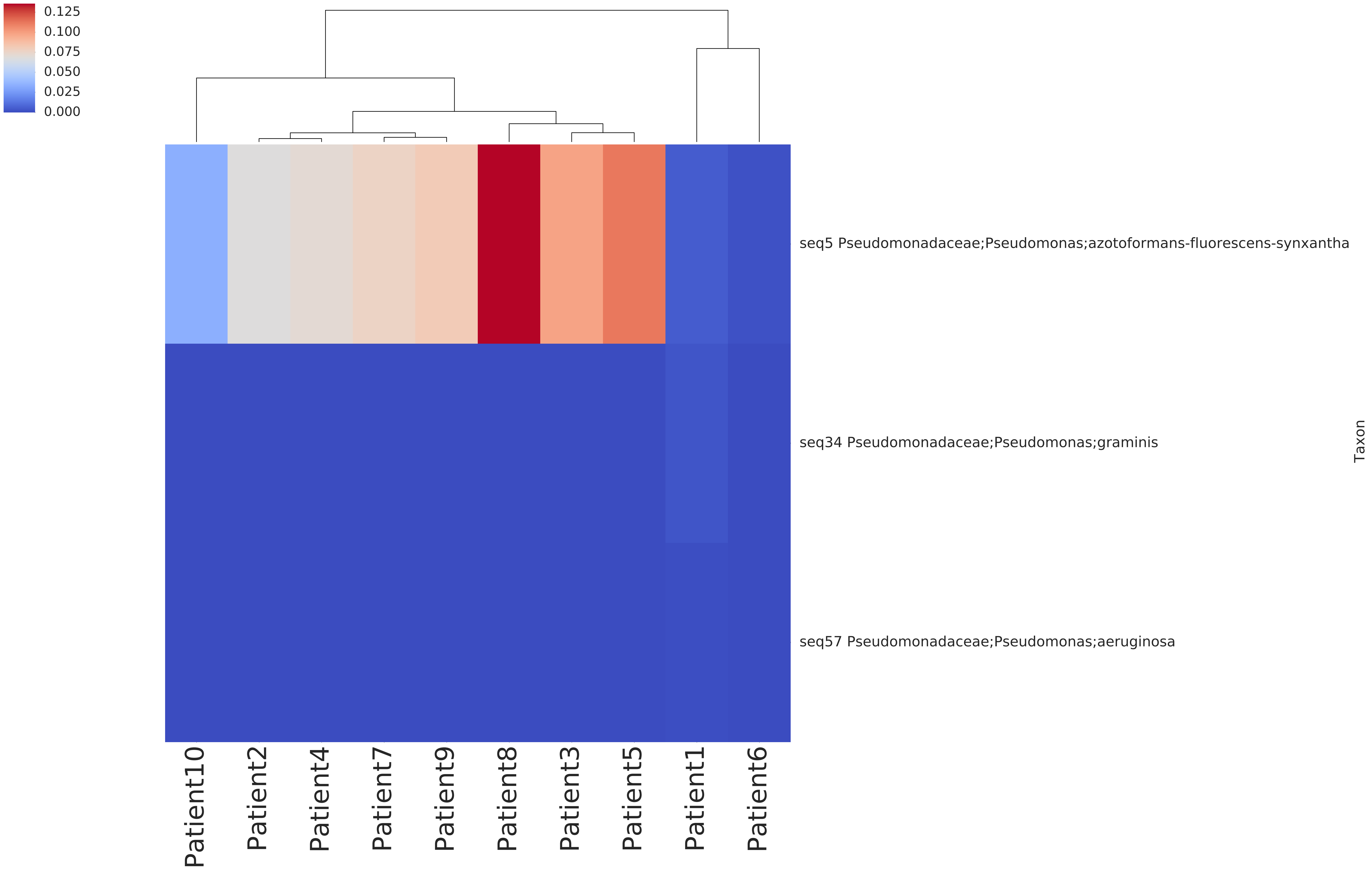
0.125
0.100
0.075
0.050
0.025
0.000
seq5 Pseudomonadaceae;Pseudomonas;azotoformans-fluorescens-synxantha
seq34 Pseudomonadaceae;Pseudomonas;graminis
seq57 Pseudomonadaceae;Pseudomonas;aeruginosa
Taxon
Patient10
Patient2
Patient4
Patient7
Patient9
Patient8
Patient3
Patient5
Patient1
Patient6
